# Unknown onset ischemic strokes in patients last-seen-well >4.5 h: differences between wake-up and daytime-unwitnessed strokes

**DOI:** 10.1007/s13760-017-0830-7

**Published:** 2017-08-12

**Authors:** Luuk Dekker, Hajo Hund, Robin Lemmens, Jelis Boiten, Ido van den Wijngaard

**Affiliations:** 1 Department of Neurology, Haaglanden Medical Center, Lijnbaan 32, 2512 VA The Hague, The Netherlands; 2 Department of Radiology, Haaglanden Medical Center, The Hague, The Netherlands; 3 Department of Neurosciences, Experimental Neurology and Leuven Research Institute for Neuroscience and Disease (LIND), KU Leuven-University of Leuven, 3000 Louvain, Belgium; 4 Laboratory of Neurobiology, VIB, Vesalius Research Center, 3000 Louvain, Belgium; 5 Department of Neurology, University Hospitals Leuven, 3000 Louvain, Belgium

**Keywords:** Ischemic stroke, Unknown onset, Wake up, Daytime unwitnessed

## Abstract

Patients with unknown time of stroke onset (UOS) represent around one-third of ischemic stroke patients. These are patients with wake-up stroke (WUS) or daytime-unwitnessed stroke (DUS), often presenting outside the time-window for reperfusion therapy. UOS patients presenting between 4.5 and 12 h after time of last-seen-well were included. Clinical and imaging characteristics were compared between WUS and DUS patients. Good functional outcome was defined as a modified Rankin scale of ≤2 at follow-up. Sixty-one UOS patients were included: 42 WUS and 19 DUS patients. Stroke severity at presentation was mild to moderate with a median National Institutes of Health Stroke Scale of 5 in WUS and 6 in DUS patients. Time between last-seen-well and presentation at the hospital was shorter in patients with DUS compared to WUS (506 vs 362 min, *p* < 0.01). CT imaging results were similar, with a median Alberta Stroke Program Early CT Score of 10 for both WUS and DUS patients. After correction for age and NIHSS at presentation, no difference in good functional outcome was found between WUS (52%) and DUS (22%). In patients with unknown onset ischemic strokes presenting between 4.5 and 12 h after time of last-seen-well, clinical and radiological features were in large part similar between WUS and DUS. The outcome in the overall cohort was rather poor despite a favorable neuroimaging profile at presentation. These findings underscore the need for clinical trials in patients in whom stroke onset time is unknown.

## Introduction

In up to 36% of cases of ischemic stroke, the exact time of onset is unknown (unknown onset stroke, UOS). About a quarter of stroke patients notice their stroke symptoms upon awakening (wake-up stroke, WUS). These patients have worse functional outcome than patients with known onset, probably due to a lack of acute treatment options [1–5].

Patients with daytime stroke onset can also present with UOS when they are unable to communicate the time of onset and it cannot be pinpointed by a witness (daytime-unwitnessed stroke, DUS) [1]. Current standard of care for acute ischemic stroke in most countries involves intravenous tissue plasminogen activator (IVT) within 4.5 h and/or intra-arterial thrombectomy (IAT) within 6 h after onset of symptoms [6, 7]. In patients with UOS, the time when they were last-seen-well (LSW) is used as a reference for time of onset of stroke, frequently exceeding the allowed time window for acute stroke treatment. As a result, a large proportion of patients with UOS are excluded from thrombolytic therapy, although treatment might be safe [8, 9].

A study comparing the characteristics between DUS and WUS patients concluded that DUS was more likely to receive acute reperfusion therapy [1]. However, DUS patients present at the emergency department (ED) earlier after LSW compared to WUS patients, often even within the 4.5-h time window for IVT. Therefore, these patients can be treated with IVT according to the guidelines.

The aim of this study was to specifically characterise patients with UOS, either DUS or WUS, presenting at the ED outside of the time window for thrombolytic therapy based on time of LSW. We compared patient demographics, clinical and neuroimaging characteristics and functional outcome between WUS and DUS. We hypothesised the frequency of early ischemic changes on neuroimaging to be more common in patients with WUS since the time between LSW and presentation might be longer compared to patients with DUS. Since all selected patients in this study presented outside the time window for IVT, we expected the functional outcome to be similar.

## Patients and methods

Patients with ischemic stroke who were admitted between January 1st, 2014, and July 31st, 2015, were extracted from our prospective stroke registry at the Haaglanden Medical Center. Patients meeting the following criteria were included:

1. Clinical diagnosis of acute ischemic stroke with unknown time of onset of stroke.
2. Last-seen-well >4.5 h (or 6 h in case of IAT-candidates) and <12 h prior to presentation.
3. National Institutes of Health Stroke Scale (NIHSS) at presentation of ≥2 points.
4. General life expectancy >90 days (exceeding time of planned follow-up).

Clinical data collected included age, gender, pre- and post-stroke modified Rankin Scale (mRS), time of LSW, time of presentation at ED, NIHSS at presentation, anterior or posterior circulation ischemia and eligibility for IVT and IAT. Patients were categorised as either WUS or DUS. Functional outcome at the 90-day follow-up was assessed using the modified Rankin Scale (mRS) which was documented in our prospective stroke registry. Good functional outcome was defined as a mRS of 0–2 at 90 days after stroke. All patients received routine clinical care in accordance with current guidelines. Eligibility for reperfusion therapy was assessed according to guidelines and current time window for acute treatment (<4.5 h for IVT, <6 h for IAT) [7, 10].

Multimodal CT imaging, including non-contrast CT and CT angiography, was evaluated by a resident in neuroradiology, who was aware of the clinical stroke symptoms, but blinded for symptom duration. The Alberta Stroke Program Early CT Score (ASPECTS), used to assess early ischemic changes, was determined on baseline CT [11]. Furthermore, collateral supply was scored on CT angiography, either being good (collaterals filling >50% of the occluded arterial territory) or poor (<50%). Both of these imaging markers are associated with functional outcome in patients receiving reperfusion therapy and could, therefore, identify patients with UOS still eligible for therapy [12].

### CT imaging protocol

Non-contrast CT was performed with contiguous 6-mm sequentially acquired axial slices. The CT angiography studies were performed on a 64 slice CT scanner (Brightspeed CT; General Electric Medical Systems, Little Chalfont, Buckinghamshire, United Kingdom) with the gantry angled to the orbitomeatal line, with 64 1-s rotations of 1.25-mm collimation and a table speed of 23 mm/s, 512 × 512 matrix, 16 cm field of view, 120 kV of variable tube current (mA) with a mean of 100 mA at the level of the circle of Willis. For CTA imaging, 50 cc of Visipaque iodine contrast material (320 mg iodine/ml) (General Electric healthcare, Little Chalfont, Buckinghamshire, United Kingdom) was injected intravenously at a rate of 6 cc/s using an automated power injector. Automated triggering of image acquisition was used at the time of contrast passage through the aortic arch, followed by a chaser bolus of saline. The CT angiographic source image data were post-processed creating coronal and axial 3-mm thick maximum intensity on a computer workstation (Advantage Workstation 4.4; Global Electronics Medical Systems).

### Statistical analysis

Clinical and radiological variables between WUS and DUS groups were compared using Chi-square tests, independent *t* tests and logistic regression. A *p* value of ≤0.05 was considered statistically significant. All statistical analyses were conducted using SPSS for Windows, version 20.0 (SPSS INC., Chicago, IL).

### Ethical considerations

The study protocol was approved by local ethical committee of the Haaglanden Medical Center and by the Medical Ethical Review Board of South-West Holland.

## Results

**Fig. 1:**
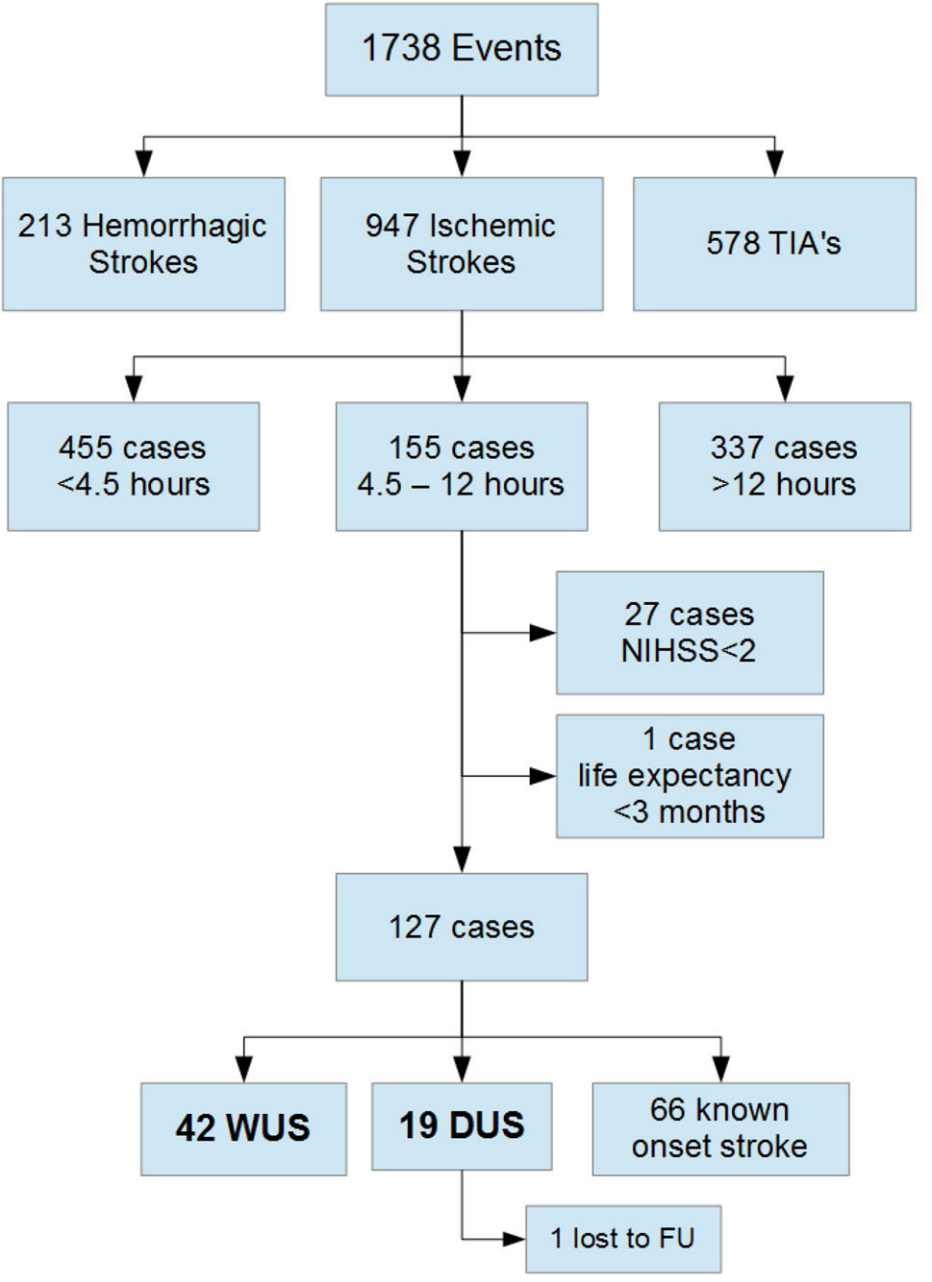
Flowchart of included patients

In 577 days, 1738 potential stroke events occurred: 213 (12.3%) hemorrhagic strokes, 578 (33.3%) TIAs and 947 (54.5%) ischemic strokes. One hundred fifty-five ischemic stroke patients (16.4%) presented at the ED between 4.5 h and 12 h after time of LSW. Twenty-seven patients were excluded because of NIHSS score <2 and one because of a life expectancy <3 months. Of the remaining 127 patients, 61 fulfilled the inclusion criteria of UOS. Of these, 19 were DUS and 42 WUS. One DUS patient was lost to follow-up due to emigration and excluded from further analysis (Fig. 1).

### Comparison of patients with WUS vs DUS

No major differences were observed in baseline characteristics although patients with DUS tended to be older compared to the group with WUS (78 vs 70 years; *p* = 0.06). As expected, the time between LSW and presentation at ED was shorter in DUS (6.0 h) compared with WUS patients (8.5 h; *p* < 0.01). Although this could suggest that patients with WUS presented later after stroke onset, no difference in early ischemic changes based on ASPECTS was identified. Collateral status was good in the majority of patients and not different between patients with WUS and DUS. At 90 day follow-up, 52% of WUS and 22% of DUS patients had good functional outcome (*p* = 0.05 for difference), defined as mRS 0–2 (Table 1). In multivariate analysis, no difference in functional outcome was found for WUS vs. DUS (*p* = 0.21) after correction for age (*p* = 0.01) and NIHSS at presentation (*p* = 0.02).

**Table 1:** Characteristics of WUS and DUS patients

	All cases (*n* = 61)	WUS (*n* = 42)	DUS (*n* = 19)	*P* value for differences DUS–WUS
Age, median (SD)	72 (15)	70 (15)	78 (14)	*p* = 0.06 (*t*)
Female sex, *n* (%)	35 (57%)	23 (55%)	12 (63%)	*p* = 0.59 (*Χ*)
Prestroke mRS ≥3, *n* (%)	4 (7%)	3 (7%)	1 (5%)	*p* = 1.0 (*Χ*)
Posterior circulation ischemia, *n* (%)	8 (13%)	5 (12%)	3 (16%)	*p* = 0.70 (*Χ*)
Time between LSW and presentation at ED in minutes, mean (SD)	461 (137)	506 (135)	362 (75)	***p*** **<** **0.01 (** ***t*** **)**
NIHSS at presentation, median (SD)	5 (3.7)	5 (3.9)	6 (3.3)	*p* = 0.61 (*t*)
Imaging characteristics for anterior circulation ischemia (*n* = 53)				
ASPECTS, median (SD)	10 (1.7)	10 (1.4)	10 (2.2)	*p* = 0.21 (*t*)
Collateral supply <50%, *n* (%)	3 (7%)	2 (7%)	1 (8%)	*p* = 1.0 (*Χ*)
Therapy eligibility				
Eligible for IVT^a^, *n* (%)	49 (80%)	34 (81%)	15 (79%)	
Eligible for IAT^a^, *n* (%)	9 (15%)	7 (17%)	2 (11%)	
IAT performed, *n*	4 (44%)	3 (43%)	1 (50%)	
Outcome				
Good outcome (mRS ≤2) after 90 days, *n* (%)	26 (43%)	22 (52%)	4 (22%)	***p*** **=** **0.05 (** ***Χ*** **)**

Significant* p* values <0.05 are shown in bold

^a^Eligibility is assessed using the currently accepted criteria excluding the criterion of time window <4.5 h (IVT) or <6 h (IAT)

(*t*) independent *t* test, (*Χ*) Chi-square test

### Eligibility for reperfusion therapy

All patients were ineligible for IVT based on presentation at ED in the time window of 4.5 h after LSW. Other exclusion criteria for IVT were only present in 12 patients (20%), similar in patients with WUS vs DUS. Nine patients (15%) were diagnosed with a large vessel occlusion >6 h after LSW. In four of these (44%) IAT was performed nonetheless (Table 1).

## Discussion

Our study shows that in patients with UOS presenting between 4.5 and 12 h after LSW, clinical and neuroimaging features were in large part similar between WUS and DUS. The rate of good functional outcome was 43% which is comparable to results in the placebo arms of IVT trials [13]. In our study, no patients were treated with IVT although other exclusion criteria were only present in 20% of patients. These findings underscore the need for assessment of reperfusion treatments for these patients in whom exact stroke onset time is unknown in randomised controlled trials, such as the DAWN, WAKE-UP and EXTEND-trials [14–16]. In addition, studies investigating expansion of time window for endovascular treatment are ongoing, perhaps providing opportunities for acute treatment in this group of patients as well [14, 17].

Imaging markers, such as small infarct core, core–penumbra mismatch, diffusion-weighted imaging (DWI)/fluid-attenuated inversion recovery (FLAIR) mismatch, perfusion/diffusion mismatch, CT perfusion–target mismatch and good collateral status, may identify patients most likely to benefit from reperfusion therapies. These selection criteria have been studied to potentially extend the time window for intravenous thrombolysis [9, 18–21] and endovascular stroke treatment [22–26]. Some of these parameters require magnetic resonance imaging (MRI) as imaging modality to identify patients who might potentially benefit from reperfusion: DWI/FLAIR mismatch and perfusion/DWI mismatch; although CT Perfusion can also be reliable on identifying patients with small core and salvageable tissue [26, 27]. In our center, CT imaging is mostly used for acute radiological assessment because this modality is more commonly used in clinical practice since it is fast, non-invasive, inexpensive, widely available and more practical than MR imaging [28]. CT Perfusion was not routinely used because until recently it was considered controversial in guiding selection of patients for stroke treatment because of equivocal results [29, 30].

Previous studies have suggested that in a large proportion of patients with WUS, stroke onset occurred only shortly before waking up, since imaging patterns were similar to known onset strokes presenting within a few hours after onset of symptoms [1, 4, 31–35]. It is, therefore, hypothesised that ischemia might be disrupting the sleeping brain, leading to awakening of the patient. These patients would probably present themselves at the ED quite shortly after onset of ischemia and thus within the treatment window of IVT. In our study, time between LSW and presentation at ED was significantly longer for WUS than for DUS patients. However, despite this 2.5-h time difference, the ASPECT score did not differ between these patients and did not reveal early ischemic changes in most patients. This suggests that onset of stroke in WUS patients might indeed be around the time of waking up potentially even caused the awakening itself. As hypothesised, no association between type of UOS (WUS vs DUS) and functional outcome was found after correction for age and NIHSS at presentation.

Our study has limitations. First, the sample size is modest with only 61 included patients. However, we had decided to only analyse patients presenting in the time window of 4.5–12 h after LSW. This enabled the specific evaluation of patients ineligible for IVT, since stroke onset time was unknown and time since LSW was more than 4.5 h, as well as the comparison of WUS vs DUS. This approach seemed appropriate to comment on findings in patients who are currently excluded from IVT. Second, some patients were treated with IAT. Although this percentage was rather low, the outcome of these four patients has potentially been modified by this intervention. Therefore, the outcome reported might not completely reflect the expected clinical course of patients with UOS ineligible for reperfusion therapy. Third, ASPECTS and collateral status were used to determine tissue status and potential benefit of reperfusion therapy. However, diffusion-weighted imaging/perfusion-weighted imaging might have been more appropriate to determine salvageable tissue in this study population.

## Conclusion

In patients with unknown onset ischemic strokes presenting between 4.5 and 12 h after time of last-seen-well, clinical and radiological features were in large part similar between WUS and DUS. Considering the favourable neuroimaging profile at presentation, a reasonable percentage of these patients might benefit from IVT. This underscores the need to identify (neuroimaging) criteria to select patients who can still benefit and assess the effect of IVT in this subgroup of patients as is currently being studied in randomised controlled trials.

## Abbreviations

ASPECTS: Alberta Stroke Program Early CT Score
DUS: Daytime-unwitnessed stroke
DWI: Diffusion-weighted imaging
ED: Emergency department
IAT: Intra-arterial therapy
IVT: Intravenous therapy
LSW: Last-seen-well
mRS: modified Rankin Scale
NIHSS: National Institutes of Health Stroke Scale
UOS: Unknown onset of stroke
WUS: Wake-up stroke
